# The “Elastic Perspective” of SARS-CoV-2 Infection and the Role of Intrinsic and Extrinsic Factors

**DOI:** 10.3390/ijms23031559

**Published:** 2022-01-29

**Authors:** Federica Boraldi, Francesco Demetrio Lofaro, Andrea Cossarizza, Daniela Quaglino

**Affiliations:** 1 Department of Life Sciences, University of Modena and Reggio Emilia, 41125 Modena, Italy; federica.boraldi@unimore.it (F.B.); francescodemetrio.lofaro@unimore.it (F.D.L.); 2 Department of Medical and Surgical Sciences for Children and Adults, University of Modena and Reggio Emilia, 41125 Modena, Italy; andrea.cossarizza@unimore.it

**Keywords:** elastin, elastase, SARS-CoV-2, COVID-19, neutrophils, NET, lung, inflammation

## Abstract

Elastin represents the structural component of the extracellular matrix providing elastic recoil to tissues such as skin, blood vessels and lungs. Elastogenic cells secrete soluble tropoelastin monomers into the extracellular space where these monomers associate with other matrix proteins (e.g., microfibrils and glycoproteins) and are crosslinked by lysyl oxidase to form insoluble fibres. Once elastic fibres are formed, they are very stable, highly resistant to degradation and have an almost negligible turnover. However, there are circumstances, mainly related to inflammatory conditions, where increased proteolytic degradation of elastic fibres may lead to consequences of major clinical relevance. In severely affected COVID-19 patients, for instance, the massive recruitment and activation of neutrophils is responsible for the profuse release of elastases and other proteolytic enzymes which cause the irreversible degradation of elastic fibres. Within the lungs, destruction of the elastic network may lead to the permanent impairment of pulmonary function, thus suggesting that elastases can be a promising target to preserve the elastic component in COVID-19 patients. Moreover, intrinsic and extrinsic factors additionally contributing to damaging the elastic component and to increasing the spread and severity of SARS-CoV-2 infection are reviewed.

## 1. Introduction

Since the dramatic spread of the severe acute respiratory coronavirus virus 2 (SARS-CoV-2)-induced pandemic, an exponentially increased number of investigations have been performed to better understand the pathogenic mechanisms, clinical manifestations as well as possible preventive and therapeutic strategies. Despite the clear evidence that lungs are primarily involved, only few reports have emphasized the impact of this infection on the extracellular compartment of the lung parenchyma. Within this context, elastic fibres represent the extracellular component exhibiting the greatest flexibility since they are capable of being extended up to 230% of their unloaded length without rupture [1]. Therefore, elastin fibres, being characterized by the Young’s modulus ranging from 30 kPa to 600 kPa, impart the elasticity that, in the lungs, allows continuous expansion and contraction of the alveolar walls. In the course of SARS-CoV-2 infection, the burst of elastolytic activities, consequent to neutrophil activation, may cause the dramatic and irreversible destruction of the elastic component, thus weakening pulmonary compliance [2].

The aim of the present review is to update and explore the current knowledge on elastic fibres, on their fate in coronavirus disease-19 (COVID-19) patients and on the therapeutic options that, limiting/inhibiting elastolytic activities, may preserve the morpho-functional properties of the lungs.

## 2. Elastic Fibres

**Figure 1:**
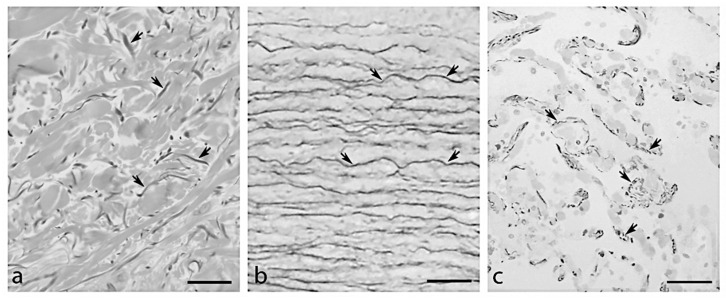
Organization of elastic fibres (arrows) observed by light microscopy in (**a**) aorta, (**b**) dermis and (**c**) lung in a subject 81 years old. Bar = 100 μm.

In the extracellular matrix (ECM), elastic fibres provide resilience and elasticity to different tissues and organs (e.g., skin, blood vessels, lungs and tendons). To accomplish tissue functional requirements, the architecture of elastic fibres is highly tissue specific, appearing in the form of elongated fibres in the skin, of sheet-like lamellae in blood vessels and of thick and thin fibres forming a branched 3D network in the lungs (Figure 1).

In the lungs, elastin is produced by mesothelial cells, airway epithelial cells, vascular and airway smooth muscle cells, endothelial cells, interstitial and lipid-laden fibroblasts and elastic fibres distributed in the respiratory parenchyma (20–30%), pulmonary blood vessels (7–16%) and the airways (7–16%) [3]. The elastin 3D organization is fundamental to guarantee that applied forces are efficiently transmitted from the alveoli to all parts of the lung [4]; therefore, defective production, altered fibre assembly and/or increased degradation are responsible for increased susceptibility to lung diseases (i.e., emphysema, *Cutis laxa*, bronchopulmonary dysplasia, chronic obstructive pulmonary disease (COPD) and acute respiratory distress syndrome (ARDS)).

Elastogenesis takes place mainly during the late foetal and neonatal stages [5]; thereafter, the elastin turnover is almost negligible [6,7]. For instance, the longevity of the elastic component in the lung parenchyma was calculated to be about 74 years, indicating that elastic fibre is a rather stable unit over the human lifespan [8].

The elastic fibre is mainly composed of elastin, whose amount varies depending on tissue (from 2% in intervertebral disks to 75% in elastic ligaments) [9,10], and of microfibrils located around the amorphous elastin as well as dispersed within it [11]. However, many other molecules, playing a role as structural components or being involved in the elastogenic process, were associated either with elastin and/or with the microfibrils or at the elastin–microfibril interface [12,13,14].

In the following sections, synthesis and assembly of elastin and of the microfibrillar scaffold will be considered separately, as the two processes are independent of each other.

### 2.1. Tropoelastin: Synthesis, Secretion, Coacervation and Cross-Linking

Tropoelastin (TE), the soluble monomeric precursor of elastin, is synthetized by elastogenic cells (e.g., fibroblasts, endothelial cells, smooth muscle cells and chondrocytes). TE is encoded by the elastin (ELN) gene, which can produce several TE isoforms by alternative splicing [15]. The TE structure is characterized by the alternation of highly hydrophobic (i.e., rich in Pro, Val, Gly, Leu, Ile and Ala residues) and of more hydrophilic domains (i.e., rich in Ala and Lys residues); the former are responsible for self-aggregation and for the tensile properties of elastin, the latter are typically involved in cross-linking. Intracellularly, TE binds to the elastin-binding protein (EBP), whose function is to hinder TE self-assembly and to avoid degradation. The TE–EBP complex, secreted at the cell surface, interacts with the elastin–receptor complex composed of neuraminidase-1, a transmembrane sialidase, and of the protective protein/cathepsin A. The binding of glycosaminoglycans to the galacto-lectin site of EBP induces conformational changes in EBP, causing the release of TE from the complex [16]. EBP is then recycled to bind to another TE molecule [17].

TE molecules undergo self-assembly through a process known as coacervation [18,19] and form spherical globules anchored to the outer surface of the cell membrane [20]. The aggregation rate, the size and other properties of the globules are modulated by several factors such as temperature, pH, ionic strength, concentration/length of individual monomers of TE and by the presence of matrix associated glycoproteins such as fibulin-4 and fibulin-5 [21,22,23,24,25,26,27]. Once assembled, TE molecules are stabilized by cross-links catalysed by lysyl oxidase (LOX) and by lysyl oxidase-like enzymes (i.e., LOXL-1 and LOXL-2) [18], which convert Lys residues into allysine (aLys) [28,29,30]. After oxidative deamination, aLys reacts with Lys and/or other aLys to form polyfunctional cross-links named desmosine and isodesmosine, two amino acids which are unique to the insoluble elastin and are necessary for elastin to be organized into a 3D structure and for the maintenance of resilient elasticity. Since elastic fibres have a negligible turnover, the presence of these amino acids in biological fluids (i.e., sputum, urine and blood) can be used as a biomarker of elastin degradation [31,32,33].

### 2.2. Microfibril Scaffold and Proteins Associated with Elastic Fibres

**Figure 2:**
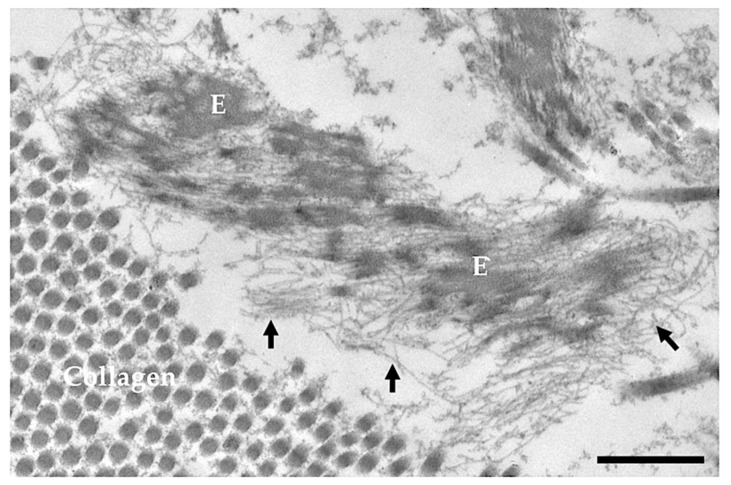
Electron microscopy image obtained from the skin of a 5-day-old healthy subject. E = elastin; arrows = microfibrils; Bar = 500 nm.

Elastin aggregates are shuttled to the microfibrils, the other major component of the elastic fibre, and are further cross-linked by LOX and/or LOXL to form complete, mature and functional elastic fibre (Figure 2). The microfibrils’ components, synthesized by mesenchymal cells (e.g., smooth muscle cells and fibroblasts), are: fibrillin-1, produced through the entire life, fibrillin-2 and fibrillin-3 (FBN), mostly expressed in embryonic tissues [34].

FBNs form microfibrillar structures with a diameter of 10–12 nm, in which individual molecules are organized in a head-to-tail arrangement producing a linear assembly. It was shown that the microfibrils’ assembly occurs at the cell surface through interaction with integrins (i.e., α5β1) and requires the presence of fibronectin [35,36] and of proteoglycans (i.e., biglycan and decorin) [37]. Similarly to elastin, FBN bundles are also stabilized by the cross-linking operated by LOX/LOXL [38,39].

In addition to the molecules reported above, fibrillin microfibrils interact with several other proteins, which play a role in elastic fibre formation. For example, latent transforming growth factor β-binding proteins support elastic fibre assembly and cell signalling [40]; microfibril-associated glycoproteins (MAGP-1 and 2) promote elastin deposition onto microfibrils and increase elastin assembly [41,42,43]; fibulins (FBL-4 and -5) facilitate elastin cross-linking by LOX/LOXL and deposition onto microfibrils [44]; a disintegrin and metalloprotease with thrombospondin type-1 repeats (ADAMTS) and ADAMTS-like proteins (ADAMTSL) are involved in microfibril assembly, adhesion and anchorage [45]; and elastin–microfibril interface-located proteins (EMILINs) are necessary for microfibril deposition onto elastic fibres [46,47]. Therefore, the development of mature and functional elastic fibres is a finely temporally and spatially regulated process requiring dozens of different proteins (for more details see also reviews [12,48,49]).

### 2.3. Elastic Fibre Degradation

It is well known that, even during the normal aging process, degradation by enzymatic (e.g., metalloproteinases) and/or chemical (e.g., U.V.) mechanisms, low/absent turnover rate of elastic fibres, altered deformability due to continuous mechanical stresses and changes in the interactions with glycosaminoglycans/proteoglycans lead to the progressive and irreversible damage of elastic fibres and to the loss of their function [50]. If these changes are amplified by the occurrence of pathologic conditions (e.g., structural deficits on a genetic basis and acute/chronic inflammatory response), the effects on tissue elasticity may have dramatic consequences [51]. Therefore, elastic fibre maintenance is the result of an accurate balance between proteolytic and anti-proteolytic activities. Elastic fibres can be degraded by endopeptidases, proteolytic enzymes that break peptide bonds in the inner regions of the polypeptide chain, which are divided into different subgroups based on catalytic mechanisms and on the presence of specific amino acid residue(s) at the active site. In general, endopeptidases work on a broad spectrum of ECM molecules (e.g., collagens, fibronectin, proteoglycans and laminin) including tropoelastin/elastin and fibrillins. In addition to being responsible for the turnover/remodelling of extracellular matrix molecules, some proteases have also been revealed within cells (e.g., nucleus, mitochondria and cytoplasm), indicating that they can also exert non-proteolytic functions (e.g., transcription factor and signal transduction) [52,53,54].

Table 1 shows the proteases that degrade TE-elastin and/or fibrillins. In addition to the enzymes reported in Table 1, some digestive enzymes, i.e., pepsin A (EC 3.4.23.1; aspartic endopeptidase), chymotrypsin (EC 3.4.21.1; serine endopeptidase), chymotrypsin-like elastase family member 2A (EC 3.4.21.71; serine endopeptidase) and trypsin (EC 3.4.21.4; serine endopeptidase), exhibit elastolytic activities [55,56,57,58,59,60]. The activity of elastases is tightly controlled by different mechanisms: transcriptional and post-translational control of gene expression; epigenetic mechanisms; cell and/or tissue specificity; precursor activation; induction of endogenous inhibitors (Table 1) [61,62,63,64]. Under physio-pathological conditions, such as in aging and in lung diseases [65], the unbalanced production of elastases and of their inhibitors leads to an altered ECM turnover. For instance, in vascular diseases it was observed that cathepsin S and K are overexpressed, whereas cystatin C is dramatically diminished, thus favouring elastin degradation [66].

**Table 1:** Peptidases acting on tropoelastin (TE)/insoluble elastin (ELN) and/or on fibrillin (FBN) and their major endogenous inhibitors.

Gene Name	Protein Name	Uniprot Accession	Major Cellular Sources	TE/ ELN	FBN	Endogenous Inhibitors
Cysteine-endopeptidases						
CTSB	Cathepsin B (EC 3.4.22.1)	P07858	Ubiquitous	[67]		Cystatin A, B, C, S [61]
CTSF	Cathepsin F (EC 3.4.22.41)	Q9UBX1	Ubiquitous	[67]		Cystatin F [61]
CTSK	Cathepsin K (EC 3.4.22.38)	P43235	Fibroblast, macrophage, osteoclast	[67]	[68]	Cystatin F [61]
CTSL	Procathepsin L (EC 3.4.22.15)	P07711	Ubiquitous	[67]		Cystatin A, B, C, D, E, M, F [61]
CTSS	Cathepsin S (EC 3.4.22.27)	P25774	Macrophage, SMC	[67]		Cystatin B, C, D, F [61]
CTSV	Cathepsin L2 (EC 3.4.22.43)	O60911	Tissue specific EC, macrophage	[67]	[68]	Cystatin E, M, F [61]
Metallo-endopeptidases						
MMEL-1	Membrane metallo- endopeptidase-like 1 (EC 3.4.24.11)	Q495T6	Fibroblast	[69]		Peptides of the opiorphin family [70]
MMP-2	72 kDa type IV collagenase (EC 3.4.24.24)	P08253	Fibroblast, macrophage, neutrophil, T-cell, VEC	[71]	[72]	TIMP-1, -2, -3, -4 [62]
MMP-3	Stromelysin-1 (EC 3.4.24.17)	P08254	EC, lymphocytes, macrophage, SMC	[71]	[72]	TIMP-1, -2, -3, -4 [62]
MMP-7	Matrilysin (EC 3.4.24.23)	P09237	EC, macrophage	[71,73]		TIMP-1, -2, -3, -4 [62]
MMP-9	Matrix metalloproteinase-9 (EC 3.4.24.35)	P14780	EC, fibroblast, macrophage, neutrophil	[71,73,74]	[72,75]	TIMP-1, -2, -3, -4 [62]
MMP-10	Stromelysin-2 (EC 3.4.24.22)	P09238	EC, macrophage, SMC	[71]		TIMP-1, -2, -3, -4 [62]
MMP-12	Macrophage metalloelastase (EC 3.4.24.65)	P39900	Lung epithelial cells, macrophage,	[76,77]	[72,75]	TIMP-1, -2, -3, -4 [62]
MMP-13	Collagenase 3 (EC 3.4.24.-)	P45452	Fibroblast, macrophage, SMC, VEC		[72,75]	TIMP-1, -2, -3, -4 [62]
MMP-14	Matrix metalloproteinase-14 (EC 3.4.24.80)	P50281	Fibroblast, macrophage, SMC, VEC	[78,79]	[72]	TIMP-1, -2, -3, -4 [62]
Serine-endopeptidases						
CELA1	Chymotrypsin-like elastase family member 1 (EC 3.4.21.36)	Q9UNI1	Lung epithelial, intestinal, and immune cells	[80]		α1-anti-trypsin [80]
CTSG	Cathepsin G (EC 3.4.21.20)	P08311	Polymorphonuclear leucocytes	[81]		α1-anti-chymotrypsin, SLPI [63]
ELANE	Neutrophil elastase (EC 3.4.21.37)	P08246	Polymorphonuclear leucocytes	[82]	[83]	α1-anti-trypsin, α2-macroglobulin, elafin [63,64]
PRTN3	Myeloblastin (EC 3.4.21.76)	P24158	Polymorphonuclear leucocytes	[84]		Elafin [64]

EC = epithelial cell; SLPI = secretory leukocyte proteinase inhibitor; SMC = smooth muscle cell; TIMP = tissue inhibitors of metalloproteinase; VEC = vascular endothelial cell.

Interestingly, enzymatic degradation is responsible not only for the progressive disruption of elastic fibres, but also for the release of soluble bioactive elastin fragments/peptides, namely elastokines, which are characterized by a GXXPG sequence (X is amino acid different to Gly). Elastokines assume a type VIII β-turn conformation, which enables binding to EBP to exert a wide spectrum of biological activities regulating [16,73]: (a) cell behaviour (e.g., proliferation and adhesion); (b) up-regulation of proteases, thus amplifying the effect of elastolysis [85,86]; (c) chemotactic activity recruiting both fibroblasts and inflammatory cells (e.g., monocytes/macrophages and neutrophils); (d) proangiogenic activity promoting endothelial cell migration and tubulogenesis through upregulation of MT1-MMP [87,88,89,90,91]; (e) osteogenic response in vascular smooth muscle cells contributing to vascular calcification [92]; (f) formation of amyloidogenic peptides [93,94,95,96]. It is worth mentioning that elastokines are also generated during physiological aging and, in the lungs, the reduced amount of functional elastin starting since the age of 35 years, causes a slowly progressive decrease in chest compliance and airspace enlargement, although not of clinical relevance [97]. Interestingly, it has been suggested that production of elastin fragments may eventually help to maintain the physiological function of the lung, allowing, in case of microbial infection, the rapid recruitment of phagocytic cells and the induction of an inflammatory response. However, even little changes in lung homeostasis and/or the exposure to environmental *noxae* can contribute to the occurrence, with age, of pathologic conditions such as COPD [98].

Moreover, proteolytic degradation and/or chemical modifications not only modify the mechanical proprieties of elastic fibres, but may also favour mineral deposition, as it has been observed in vascular as well as in pulmonary diseases [99]. It has already been demonstrated that elastin, due to its structural characteristics, can bind, with high affinity, Ca^2+^ ions through its neutral carbonyl groups [100]. Moreover, ELN fragmentation generates a higher number of nucleation sites, which bind to Ca^2+^ ions, that, in turn, interact with phosphate, markedly increasing the calcification process [101]. Another aspect that deserves to be considered is that elastin degradation leads to the release of molecules normally sequestered in the extracellular milieu activating, for example, TGF-β/bone morphogenic protein signalling pathways [102,103].

## 3. SARS-CoV-2 Infection

SARS-CoV-2, the seventh member of the single-strand enveloped RNA Coronaviruses family, infects mammalian and avian species causing the Coronavirus Disease (COVID-19), which affects the respiratory, gastrointestinal and central nervous system. The pandemic spread of COVID-19 since December 2019 rapidly became, and remains, of global public health concern [104]. The clinical features and severity of COVID-19 vary significantly among individuals, based on multiple factors, such as the presence of genetic polymorphisms [105], age, environmental factors and associated comorbidities such as diabetes, hypertension, cardiovascular disease, chronic kidney disease, cancer and obesity [106,107]. Although a consistent number of patients may not require hospitalization, severe cases suffering from systemic inflammation, pneumonia and hypoxemia may become critically ill with complex organ failures which may lead to death [108]. In particular, ARDS and respiratory failure are the leading causes of death and emphasize the key role of lung involvement in hospitalized COVID-19 patients [109,110].

SARS-CoV-2 enters within target cells through the angiotensin-converting enzyme 2 receptor (ACE2r), which is highly expressed in the ciliated cells of nasal and bronchial epithelia and in the type II alveolar cells. Moreover, ACE2r is also present in the heart, liver and kidney, and its expression has been suggested to be modulated by comorbidities and by COVID-19 risk factors (e.g., age, COPD, diabetes, tobacco smoke and hypertension), although contradictory findings were frequently reported, thus preventing the establishment of a true relationship with SARS-CoV-2 infection [111]. Recently, it was proposed that lectins and phosphatidylserine receptors may represent additional host entry factors, however, since they were not found in association with SARS-CoV-2 infection in the absence of ACE2r, these molecules should more likely be considered as “attachment factors” [111].

The interaction of SARS-CoV-2 with ACE2r is mediated by the spike protein that consists of two subunits: S1 that binds to ACE2r and S2 that anchors the S protein and contributes to create a fusion pore on the cell membrane allowing the virus to enter. It is important to note that the spike protein works only if proteolysis takes place at the two cleavage sites (i.e., at the S1–S2 junction and within the S2 subunit) [112,113,114].

Early infection is characterized by an extensive engagement of the immune system with changes in T and B cells [115,116], alterations in the formation of virus-specific lymphocytes [117], mitochondria alterations and oxidative stress [118]. Severe COVID-19 pathophysiology is characterized by altered neutrophil quantity, phenotype and functioning in the blood as well as in the lungs [119], where neutrophils contribute to the proteolytic damage of tissue elasticity through a disproportionate release of virus-induced neutrophil extracellular traps (NETs) [120]. Interestingly, it has been demonstrated in experimental animal models that the pulmonary vascular bed is the major site of granulocyte margination, thus accounting for the hypothesis that 90% of total blood granulocytes resides in the lungs [121]. Moreover, several neutrophils can remain immobilized within pulmonary capillaries and their amount is markedly increased when neutrophils are primed [122,123]. It is therefore conceivable that neutrophils, upon their activation during COVID-19, release, mainly in the airways, the great majority of preformed inflammatory mediators [124]. Moreover, granule-derived peptides and proteolytic enzymes can interact with threads of chromatin to form the NETs serving as an additional defence of the innate immune system [125]. Neutrophil elastase (NE)/DNA complexes in NETs may also play a role in the development of acute haemorrhagic or thrombotic plaque complications [126]. Therefore, in patients severely affected by COVID-19, the accumulation of intravascular NETs interferes with the plasminogen proteolytic pathway inducing platelet trapping, fibrinolytic collapse and microvascular occlusion leading to multi-organ failure [124]. Moreover, it has also been demonstrated that NETs, directly killing epithelial and endothelial cells, can contribute to tissue damage [127], that, in COVID-19 patients, is followed by an aberrant healing process that may lead to pulmonary fibrosis [128].

At present, there is a serious concern with post-COVID-19 sequelae, and several studies were performed and are still ongoing for a better long-term management of COVID-19 patients [129,130]. It is worth mentioning that critically ill patients may experience reduced lung functions and dyspnoea due to disruption of the normal lung architecture [131,132]. The extent of tissue damage is highly variable and may exert morpho-functional consequences which are often fully recovered, but that may also lead to a permanent, although stable, injury or, in a small group of patients, may continuously progress, thus posing, in the most dramatic cases, the option of lung transplantation as the only life-saving therapeutic strategy [133]. Collagen deposition and the development of fibrosis were observed in hospitalized COVID-19 patients with a percentage ranging from 19% [134] to 72% [135]. Consistently, a proteomic study, performed on pulmonary tissues after SARS-CoV-2 infection, showed a differential expression of several ECM proteins and suggested that these changes can be associated to lung remodelling and fibrosis [136]. In agreement with these findings, histopathological analyses on lungs from COVID-19 patients revealed a loss of elastic fibres and an increase in collagens type I and III [130,137]. The pulmonary fibrosis may start early during ARDS, however, it is more common in patients with longer durations of intensive care unit (ICU) stay (e.g., more than 3 weeks) [128,138]. The mechanisms associated with the development of fibrotic changes are still poorly understood and are probably multifactorial; moreover, the relatively short duration of reported follow-up is probably not sufficient to establish, clearly and unambiguously, the functional consequences and the patho-mechanisms characteristics of tissue damage [139]. For instance, it has been suggested that collagen deposition starts when: (i) a mesenchymal transition is induced in epithelial and endothelial cells upon SARS-CoV-2 infection [140,141]; (ii) fibroblast and myofibroblasts proliferate to repair damaged/necrotic areas [142]; (iii) the expression of pro-fibrotic cytokines, such as transforming growth factor-beta (TGF-β), is triggered by angiotensin II production [130,143]; (iv) ventilator-induced lung injury and oxygen toxicity significantly contribute to oxidative stress damage [144,145]. All these changes sustain the rationale of a number of ongoing clinical trials aiming to prevent/limit the progression of post-COVID-19 lung fibrosis [139].

In the lungs, collagen and elastin represent the most prevalent structural elements assuring the airways’ functional properties, and in the interstitium of alveolar walls more than 40% of collagen and elastic fibres are highly interconnected with each other, indicating that a strong relationship exists between morphological organization of the extracellular matrix and pulmonary mechanical properties [146]. Since prolonged/severe inflammation can alter the organization of these components, it is also important to investigate the role and the fate of the elastic component after SARS-CoV-2 infection to better understand post-COVID-19 symptoms [147].

## 4. Elastic Fibres and Elastases in SARS-CoV-2 Infection

In COVID-19 patients, neutrophils are the major source of elastases, which are stored in cytoplasmic granules and are released upon neutrophil activation as part of an inflammatory response to viral infection [148]. However, the role of NE is more complex than initially thought. Neutrophil-derived elastolytic enzymes are fundamental for SARS-CoV-2 to enter target cells. Membrane fusion is in fact favoured by the cleavage of the viral spike protein by neutrophil-derived proteases such as neutrophil elastases, cathepsins, furin and transmembrane serine protease (TMPRSS2 and TMPRSS11A) [149]. To further sustain the role of elastases in SARS-CoV-2 infections, there are a number of mutations that, affecting the SARS-CoV-2 spike protein (e.g., p.Asp614Gly, p.Thr716Ile and p.Ser982Ala), can increase COVID-19 transmissibility and favour the entry ability of the virus by introducing new proteolytic cleavage sites for elastases [150,151]. Interestingly, in the presence of α1-antitrypsin (AAT) deficiency, the host-cell entry of the Asp614Gly variant of the virus is further enhanced due to the delayed NE inhibition and the boosted activation of the spike protein [152].

NE also represents a key player in NETosis. In particular, NE degrades actin cytoskeleton and translocates to the nucleus where it cleaves histones leading to chromatin relaxation and DNA decondensation, followed by nuclear membrane disruption and plasma membrane disintegration with the release of NETs in which elastases are entrapped, remaining active even when NETs are exposed to endogenous proteinase inhibitors [153,154]. Alternatively, activated neutrophils can degranulate, releasing their content in the extracellular space and may also expel nuclear chromatin while remaining alive [127,155]. In the extracellular milieu, elastolytic activities of NE as well as of cathepsin L and of MMP-9 produced by alveolar macrophages, are responsible for the lung tissue destruction that limits airflow and therefore contributes to pulmonary complications [156,157]. Notably, the absent turnover of the elastic component markedly worsens the consequences of the enhanced degradation driven by high levels of elastolytic enzymes associated with the inflammatory burst, and these events cause loss of recoil and failure of the elastic airway support. Moreover, the altered balance between protease/antiprotease activities can favour the release of elastokines, which in turn induce the additional expression of proteases and sustain the inflammatory response as well as the recruitment of mesenchymal and inflammatory cells [158].

Consistently, in COVID-19 patients, plasma levels of NE were shown to be 10–20 times higher compared with controls [125]. Similarly, desmosine and isodesmosine (DES), two biomarkers for elastin degradation, were also significantly increased in patients and DES levels correlated with the amount of IL-6, suggesting “a key link between inflammation and pulmonary/vascular tissue damage in COVID” [159]. It is therefore of particular interest to observe that elastases can contribute to the destruction of extracellular matrix components of the lung parenchyma (e.g., collagen, elastin, glycosaminoglycans and fibronectin) [160] and can also play a promotive role in pulmonary fibrosis [161]. NE, in fact, was demonstrated to up-regulate the expression of Notch1 that elicits myofibroblast differentiation of alveolar epithelial cells via a TGF-β–Smad3 signalling pathway [162]. Moreover, Notch1 activation directly stimulates the expression of α-smooth muscle actin (α-SMA) to induce myofibroblast differentiation [163]. In line with these data, there are studies demonstrating that NE, TGF-β and α-SMA are significantly up-regulated in COVID-19 patients [130].

## 5. Intrinsic and Extrinsic Factors Impairing Elastic Fibre Homeostasis and Their Impact on SARS-CoV-2 Infection

### 5.1. Aging

With age, elastin undergoes structural and functional alterations due to: (i) repeated stretching and mechanical deformation; (ii) progressive degradation by altered balance between proteolytic/anti-proteolytic activities; (iii) chemical modifications as consequence of exposure to environmental *noxae*, including oxidative stress and increased glycation, thus leading to progressive loss of tissue elasticity [51]. As demonstrated by structural studies, glucose modifies the proportion of beta-sheets, beta-turns and alpha-helices present in the elastin molecule. These conformational changes may lead to a more rigid structure [164,165] and to alterations of the viscoelastic properties, thus increasing the stress relaxation response [166].

Evidence was provided that children, at least before the introduction of the vaccination plans, were less affected by SARS-CoV-2 compared with adult or old individuals [167], that the percentage of patients increases proportionally to their age and that there is an age-dependent increase in severe symptoms which require hospitalization [168]. Since aging is also associated with comorbidities, the direct contribution of aging in higher rates of both mortality and morbidity remains unclear. Therefore, several models were proposed to simulate the dynamics of virus spreading [168,169,170]. Analysis of data from three European countries (i.e., Italy, Spain and United Kingdom) clearly indicates an age-dependent susceptibility to SARS-CoV-2 infection [168], thus suggesting that in countries with higher rates of elderly, the SARS-CoV-2 virus spreads more rapidly and might be associated with the occurrence of clinical manifestations [169].

The age-depended increased susceptibility to COVID-19 is in line with several findings involving, for instance, the elastic component and the whole extracellular milieu, the efficiency of the immune response and the redox homeostasis. Several reports have already underlined the role of advanced glycation end products (AGEs), produced by glycation of amino acids, lipids and DNA in the development of ‘‘inflammaging’’ and of comorbidities, which contribute to several aspects of COVID-19 pathogenesis in the elderly [171,172]. AGE receptors (RAGE), highly expressed by alveolar epithelial cells and macrophages, were reported to increase phagocytic activity and to be involved in NET formation, thus playing a central role in the lung inflammatory cascade of events caused by SARS-CoV-2 [173]. As a consequence of severe COVID-19, tissue damage may dramatically affect the extracellular compartment and RAGE, sustaining the elastolytic activities and the repair processes contributing to pulmonary fibrosis, which is more often reported in aged patients [174].

Since several polymorphisms of the RAGE gene (AGER) were reported to either promote or reduce the susceptibility to lung diseases as COPD and ARDS, it is conceivable to hypothesize that different AGER variants might differentially predispose patients to COVID-19 comorbidities and can modulate the outcome of SARS-CoV-2 infection, thus suggesting that RAGE represent potential therapeutic targets [175].

### 5.2. Oxidative Stress

It is well known that oxidative stress plays a key role in the pathogenesis of several age-related diseases (e.g., cardiovascular and pulmonary diseases) [176,177]. Oxidants derive both from internal (e.g., mitochondrial respiration and inflammatory cells) and external mechanisms/factors (e.g., smoke and UV exposure) and can modify several biological macromolecules (i.e., protein, lipids and nucleic acid). In this context, reactive oxygen (ROS) and nitrogen (RNS) species play a crucial role in elastic fibre degradation and/or assembly. Furthermore, elastin, as a typical long-lived protein, undergoes non-enzymatic post-translational modifications such as glycation and carbamylation. The latter is directly associated with oxidative-stress damage and is responsible for the progressive age-dependent increase in elastic fibre stiffness [80]. In addition, Umeda et al. [31] isolated and identified two new dihydrooxopyridine cross-links from bovine aortic elastin: oxodesmosine and isooxodesmosine, which derive from the metal-catalysed oxidation of desmosine and isodesmosine. These modifications induce the solubilization of insoluble elastin. Another in vitro study showed a synergistic effect of H_2_O_2_ and elastases on elastic fibre: an oxidant pre-treatment can in fact reduce the stability of elastic fibres enhancing their susceptibility to elastase-mediated degradation [178]. Moreover, it was demonstrated that ROS and RNS influence TE assembly [179]. For instance, peroxynitrite and hypochlorous acid, produced by leucocytes, increase in vitro TE coacervation but reduce cross-linking and the interactions with other proteins (e.g., fibulin-4 and -5) necessary for elastic fibre assembly. Therefore, oxidative damage can contribute to the abnormal structure and function of elastic fibres in both physiological and pathological conditions. Although further in vivo studies are necessary to understand the precise role of oxidative stress in elastic fibre degradation, these data support the idea that changes in oxidative stress could, at least in part, be responsible for reduced cross-linking and contribute to elastic fibre dysfunction. It is worth mentioning that recent studies indicated that altered redox balance (the ratio between prooxidants and antioxidants) also plays an important role in mild forms of SARS-CoV-2 infection [180,181].

In addition, ROS represent important signals, which may lead to NETosis. In particular, ROS generated by NADPH oxidase stimulate myeloperoxidase (MPO), allowing the release of NE from the azurosome present within granules [154]. Neutrophil elastase then translocates to the nucleus contributing to the proteolytic disruption of chromatin required for NET formation [182]. In contrast to histone-DNA and MPO-DNA, NE is considered an independent predictor of multi-organ damage in COVID-19 patients, thus underlining the role of elastases in SARS-CoV-2 infection [182].

Beside oxidation, oxidative stress also favours degradation and fragmentation of ECM components (e.g., elastin, heparan sulphate and hyaluronan), which are important factors for triggering lung inflammation and causing airway enlargement. ECM fragments (e.g., elastokines) exert an active role in the recruitment of inflammatory cells that can further increase the respiratory burst (ROS production) upon neutrophil activation. Prevention of the oxidative fragmentation of ECM components is performed by antioxidant enzymes such as extracellular superoxide dismutase [183], which is highly expressed in lungs and vessels [184] and, being located in the ECM, acts as a superoxide anion scavenger, thus counteracting the oxidative stress and damage of matrix components.

### 5.3. Smoke

Cigarette smoke (CS) is a complex mixture of over 8000 chemical substances (e.g., aromatic amines, pyridine, carbon monoxide and ammonia), many of which are toxic/carcinogenic for humans [185]. Moreover, CS representing an exogenous source of ROS and RNS induce oxidative stress in association with the inhibition of antioxidant systems, upregulation of elastases (e.g., MMP-1, -3 and NE) and down-regulation of their inhibitors (e.g., α1-antitrypsin) [186,187,188,189]. Morphometric and immunohistochemical analyses performed on skin biopsies demonstrated that two principal components of elastic fibres (i.e., elastin and microfibrils) were altered in smokers compared with non-tobacco consumers. In particular, smokers showed an increased number of elastic fibres due to an enhanced degradation/fragmentation process [187,190]. In the lungs, the CS-induced imbalance between elastases/elastase inhibitors and oxidant/antioxidant levels can destroy elastin-rich structures leading to the onset of COPD, consisting of emphysema and chronic obstructive bronchitis [191,192]. Some components of CS can react with plasma and ECM proteins to form AGEs, which accumulate in tissues/organs [193]. AGEs efficiently bind to RAGEs, whose expression is markedly increased in smokers, predisposing them to COPD [175]. The activation of RAGEs alters several cell-signalling pathways (e.g., mitogen-activated protein kinases and nuclear factor kappa-B), which are involved, for example, in different inflammatory and immune processes, leading to increased expression of chemokines (e.g., monocyte chemoattractant protein-1) and proinflammatory cytokines (e.g., tumor necrosis factor-α and interleukin-6) [175,194,195,196].

Since CS was involved in the pathogenesis of various lung diseases characterized by inflammation and pulmonary functional decline, CS was also expected to be involved in the pathogenesis of COVID-19. Surprisingly, several investigations reported contradictory results. For instance, Hippisley-Cox and co-workers [197] reported that smoking was associated with a lower risk of severe COVID-19. Following a number of studies, a “smoker’s paradox” was put forward [198] due to the fact that nicotine, a cholinergic agonist and pro-inflammatory cytokine inhibitor, might lower the amount of ACE2r, thus claiming protective and/or therapeutic effects of smoking/vaping in relation to COVID-19 [199]. Even though the biological effect of nicotine per se was demonstrated in experimental models, contradictory findings were still reported, such as the over-expression, for instance, of ACE2 in smokers [200]. By contrast, in the OpenSAFELY study, current smoking was associated with higher risk for COVID-19 death [201], and these findings were recently supported by an observational and Mendelian randomization study on a UK biobank cohort [202]. Nevertheless, it is important to underline that the chronic use of tobacco cigarettes and e-cigs weakens the respiratory system performance, worsening the inflammatory response and the tissue damage resulting from SARS-CoV-2 infection. Therefore, it is still important to fight smoke addiction to counteract the adverse health effects of smoking and in particular the harmful effects on elastic structure maintenance [203]. In vivo studies in the rat model showed that a relatively short-term exposure to cigarette smoke significantly enhances the emphysematous effects of exogenously administered elastolytic enzymes [204].

Since Janoff [205] reported an increased level of NE in the lung fluids of smokers altering the homeostatic balance between endogenous lung proteases and protease inhibitors, it is conceivable that smoking, negatively interfering with the elastic microenvironment, has an additional negative impact on the progression and severity of SARS-CoV-2 infection.

### 5.4. Vitamin K

Vitamin K is either introduced with food (phylloquinone or vitamin K1) or synthesized by the intestinal microbiota (menaquinone or vitamin K2), both are required for the γ-glutamyl carboxylation of vitamin K-dependent proteins (i.e., coagulation factors II, VII, IX, X, matrix-gla protein, Gas6 and protein S) [206]. Consistently, vitamin K deficiency results in altered carboxylation, increased calcification of soft connective tissues and enhanced thrombogenicity [207]. Given the involvement of elastin and of the coagulation system in COVID-19 patients, it was hypothesized that vitamin K may play a role in the disease. A recent study demonstrated that vitamin K insufficiency is present in hospitalized COVID-19 patients and that low vitamin K levels are associated with several comorbidities which worsen the clinical outcome of these patients [208]. Since the elastic component, being calcified and/or fragmented, appears compromised in aging and in age-related diseases (e.g., diabetes, hypertension, cardiovascular and pulmonary diseases) [101,209], it can be hypothesised that vitamin K deficiency contributes to the dysfunction of elastic fibres and facilitates their proteolytic degradation after SARS-CoV-2 infection, thus increasing the severity of clinical complications [159].

### 5.5. Pollution

It is well known that air pollution (e.g., PM_2.5_ and PM_10_ particulates, nitrogen oxides, ammonia, ozone, sulphur dioxides, carbon monoxide and volatile organic substances) is associated with respiratory diseases as interstitial lung diseases and COPD through mechanisms involving oxidative stress and inflammation [210]. In general, particulate inhalation, depending on size, 3D-structure and chemical composition, can reach deep airways, where particles are engulfed by phagocytic cells (i.e., neutrophils and macrophages) that become activated and release cytokines as well as proteolytic enzymes. These events trigger an inflammatory response leading to cell recruitment, damage of the lung parenchyma through degradation of the elastic component and progressive increase in collagen deposition [211], thus accelerating the pulmonary functional decline and representing a favourable environment for SARS-CoV-2 infection. Consistently, the prevalence of COVID-19 and high levels of morbidity and mortality were observed in more polluted regions, although confounding factors such as age, population density and pre-existing comorbidities cannot be excluded [212]. It was shown that viruses can be adsorbed by PMs, diffused into the atmosphere and transported over long distances [213]. Moreover, it was shown that prolonged exposure to PM_2.5_ can increase oxidative stress and inflammation, whereas PM_10_ particles may act as carriers of droplet nuclei favouring SARS-CoV-2 transmission [214].

### 5.6. Mechanical Stress

Interactions between ECM components and between ECM and cells have several regulatory effects on cellular behaviour as well as on the biomechanical and functional properties of the lungs [215]. It is well known that elastin cleavage produces an irreversible structural damage, lowering the ability of tissue to cope with the effect of mechanical forces. For instance, when an elastic fibre is structurally damaged, the load is increased on the surrounding fibres, which become more susceptible to further damage/break [2]. For instance, results reported by Jesudason and co-workers [216] suggested that, within a tissue, enzyme activity can be locally regulated by the capability of the ECM to transmit macroscopic forces, which in turn accelerate the enzymatic destruction of the alveolar walls. These findings indicate that proteolytic degradation not only alters the morpho-functional properties of elastic fibres as well as their interactions but can also act in synergy with mechanical forces.

Interestingly, it was demonstrated that mechanical ventilation may pose an additional risk of ventilator-induced lung injury arising from abnormalities of pressure or volume setting, thus affecting the biomechanical properties of the lungs [174]. Since ICU treatments are required in 5–12% of COVID-19 patients, due to the severity of the disease, the biomechanical consequences on ECM organization and functional compliance may exert long-term effects. Consistently, it has been reported that patients who develop post-COVID-19 lung fibrosis are those who suffered from extensive lung involvement and required mechanical ventilation with high oxygen concentration [138]. Moreover, in these circumstances, oxygen-derived free radicals can further alter the pulmonary epithelium, and it was demonstrated, in an experimental model, that exposure to 60% oxygen enhances the effects of elastase injury and contributes to the disruption of the interactions between ECM components [217].

## 6. Elastase Inhibitors: Any Valuable Perspective in SARS-CoV-2 Infection?

Elastases represent a critical factor for viral entry into cells as well as for inducing matrix destruction, hypertension, thrombosis and vasculitis [151]. As there are several elastase inhibitors, some of them, already proposed and/or used to treat inflammatory lung diseases [218], may be considered within the context of multitarget therapeutic approaches in SARS-CoV-2 affected patients which are characterized by excessive release of elastolytic enzymes (Table 2).

**Table 2:** Elastase inhibitors and their use as therapeutic molecules.

Compound	Activity	Applications
Sivelestat (ONO-5046)	Selective, reversible and competitive neutrophil elastase inhibitor without effects on other proteases.	Approved for acute respiratory syndromes and proposed for COVID-19 [124].
Roseltide	A plant derived peptide acting as a neutrophil elastase inhibitor.	Proposed for airway inflammatory diseases [219].
Lonodelestat (POL6014)	A macrocycle based on the protein epitope mimetic technology acting as a potent and selective neutrophil elastase inhibitor.	Proposed for chronic inflammatory conditions and in phase 2 trial for patients with cystic fibrosis [220].
Alvelestat (MPH966)	Neutrophil elastase inhibitor.	Proposed for bronchiolitis obliterans syndrome, emphysema, COPD and in phase 2 trial for COVID-19 patients [182,221].
Brensocatib (INS1007)	Selective, competitive and reversible cathepsin C inhibitor that reduces neutrophil elastase activity.	In phase 3 trial for COVID-19 patients [222]
Prolastin	α1-antitrypsin.	Approved for self-administration AAT therapy to preserve functional lung tissue in AAT deficiency, COPD and proposed for COVID-19 [223].
Elafin	Endogenously synthesized protein containing domains with antiproteolytic properties (i.e., vascular elastase).	Proposed for the treatment of inflammatory vascular, systemic and pulmonary diseases as COPD [224].
Secretory leucocyte protease inhibitor (SLPI)	Unglycosylated natural protease inhibitor with a additional role as NET modulator.	Proposed for COPD, chronic lung diseases [224,225].

One of the most investigated and commercially available elastase inhibitor is sivelestat, a selective, reversible and competitive neutrophil elastase inhibitor, that, in different models of lung injury [226] and in patients with ARDS [227,228], was shown to improve pulmonary function and to shorten the duration of mechanical ventilation time and length of ICU treatments, possibly through inhibition of the overstretch-induced signalling pathway (i.e., phosphorylation of c-Jun NH2-terminal kinase) and neutrophil chemotaxis [226]. Moreover, since elastases contribute to spike protein activation, thus favouring virus entry into target cells, treatment with sivelestat, by inhibiting NE, may exert a dual effect by lowering the damaging effect on the pulmonary connective tissue and limiting the virus from spreading [229].

An almost completed depletion of proinflammatory elastolytic enzymes was described in neutrophils from patients with Papillon-Lefèvre syndrome (PLS), a disease characterized by cathepsin C (CatC) deficiency. CatC, by removing N-terminal dipropeptides, activates most tissue-degrading elastase-related serine proteases (elastase, cathepsin G, proteinase 3 and NSP4) [230], and therefore represents a potential therapeutic target to counteract protease-driven tissue degradation in inflammatory diseases and plausibly in COVID-19 patients [222]. Impairing neutrophil elastases and/or the release of NETs could have a positive impact of lung tissue conservation, but, at the same time, can decrease the capabilities of host innate immunity. Interestingly, it was observed that cells from PLS patients are unable to produce NETs, but, unexpectedly, these patients do not exhibit signs of immunodeficiency or of recurrent viral infections [230], thus supporting the hypothesis that a pharmacological inhibition of CatC activity may represent an attractive therapeutic strategy to safely and efficiently regulate elastase-related serine proteases and to avert the irreversible pulmonary failure in COVID-19 patients [222].

The level of NE is regulated by AAT that is mainly produced by the liver, but it is also expressed by neutrophils, macrophages and pulmonary alveolar cells [231]. AAT acts as an inhibitor of inflammatory molecules (e.g., IL-8, TNF-α) and of proteases involved in the pathophysiology of COVID-19 (e.g., elastase, TMPRSS2 and ADAM17) [232]. Since TMPRSS2-mediated SARS-CoV-2 entry into host cells, AAT was demonstrated to be capable to inhibit SARS-CoV-2 entry, and therefore can represent an anti-COVID-19 treatment [233,234]. In addition, inhibition of ADAM17 can modulate the ACE2 cleavage and the “cytokine storm” typical of COVID-19 and the risk of vascular hyperpermeability, multiorgan failure and death [235]. Moreover, since ADAM17 is overexpressed in diabetic patients, the high risk of COVID-19 complications in these patients can be due to the inhibition of AAT activity by diabetic-dependent glycosylation [236].

AAT plasma levels can normally increase 3- to 5-fold in the course of systemic inflammation and/or infection, however, AAT was observed to be present at low levels in severe cases of COVID-19 [237]. In agreement with these findings, Vianello and Braccioni [238] showed that, in Italy, there was a geographic co-localization between individuals with AAT deficiency and the number of COVID-19 cases. Interestingly, AAT serum levels can be modulated by genetic variants as well as by cigarette exposure, oxidative stress and pollution, and these changes may have a significant relevance in lowering the protective role of AAT during COVID-19 [234,237]. Since AAT irreversibly inhibits serine proteases and elastases, but may also exert anti-viral, anti-TMPRSS-2, anti-inflammatory, anti-thrombin, anti-NETs and antiapoptotic activities, AAT represents an additional candidate for the treatment of COVID-19 [234]. Within this context, elafin and the secretory leucocyte protease inhibitor (SLPI), being a member of the serine protease inhibitor family, which also includes AAT, may be used to potentiate the action of AAT or to act as substitutes in case of AAT deficiency. Interestingly, SLPI has an additional role as an anti-inflammatory agent by lowering the secretion of pro-inflammatory cytokines, preventing neutrophil infiltration and regulating the activity of NK-kB [239].

## 7. Conclusions

Elastin is the major constituent of elastic fibres and one of the longest-lived and metabolically stable proteins in the body; nevertheless, it is progressively degraded during aging and as the result of inflammatory reactions. These events, further enhanced by several intrinsic and extrinsic factors (e.g., aging, oxidative stress, smoke, pollution, vitamin K and mechanical stress), may cause loss of tissue elastic recoil as well as the activation of signalling pathways driven by the release of elastokines. The massive recruitment and activation of neutrophils and the consequent release of ROS and of elastolytic enzymes, which take place in severely affected COVID-19 patients, are responsible for their pulmonary loss of function. However, the irreversible damage that affects the elastic component and the failure of the body to repair and to reconstitute tissue elasticity were only hardly addressed. In this scenario, the presence of several elastase inhibitors, already tested for other lung diseases, deserves special attention and may open new perspectives in the wide spectrum of treatments aiming to counteract the entry of SARS-CoV-2 into target cells, as well as to preserve the elastic component to avoid the long-term consequences and the persistence of symptoms in COVID-19 survivors.
